# Egypt's Red Sea coast: phylogenetic analysis of cultured microbial consortia in industrialized sites

**DOI:** 10.3389/fmicb.2014.00363

**Published:** 2014-08-11

**Authors:** Ghada A. Mustafa, Amr Abd-Elgawad, Alyaa M. Abdel-Haleem, Rania Siam

**Affiliations:** ^1^Biotechnology Graduate Program, Biology Department and YJ-Science and Technology Research Center, American University in CairoNew Cairo, Egypt; ^2^Tourism Development Authority, Ministry of TourismCairo, Egypt

**Keywords:** Red Sea, Taba/Eilat, 16S rRNA, mangrove, oil/hydrocarbons, Vibrios, *Clostridium botulinum*, Solar lake

## Abstract

The Red Sea possesses a unique geography, and its shores are rich in mangrove, macro-algal and coral reef ecosystems. Various sources of pollution affect Red Sea biota, including microbial life. We assessed the effects of industrialization on microbes along the Egyptian Red Sea coast at eight coastal sites and two lakes. The bacterial communities of sediment samples were analyzed using bacterial 16S rDNA pyrosequencing of V6-V4 hypervariable regions. The taxonomic assignment of 131,402 significant reads to major bacterial taxa revealed five main bacterial phyla dominating the sampled sites: Proteobacteria (68%), Firmicutes (13%), Fusobacteria (12%), Bacteriodetes (6%), and Spirochetes (0.03%). Further analysis revealed distinct bacterial consortia that primarily included (1) marine *Vibrio* spp.—suggesting a “marine *Vibrio* phenomenon”; (2) potential human pathogens; and (3) oil-degrading bacteria. We discuss two divergent microbial consortia that were sampled from Solar Lake West near Taba/Eilat and Saline Lake in Ras Muhammad; these consortia contained the highest abundance of human pathogens and no pathogens, respectively. Our results draw attention to the effects of industrialization on the Red Sea and suggest the need for further analysis to overcome the hazardous effects observed at the impacted sites.

## Introduction

The Red Sea possesses a unique geography, as it is almost entirely locked by land, and its ecosystems are diverse, including mangrove, macro-algae and coral reefs (Alkershi and Menon, 2011). The Red Sea encompasses two gulfs, the Gulf of Suez and the Gulf of Aqaba, in addition to the Red Sea proper. The Gulf of Suez is entirely bordered by Egypt, while the borders of the Gulf of Aqaba are shared among four countries: Egypt, Israel, Jordan, and Saudi Arabia. The Red Sea proper is bordered by six countries: Egypt, Sudan, Eritrea, and Djibouti on the western shore and Saudi Arabia and Yemen on the eastern shore.

Severe pollution of the Red Sea has been reported multiple times from Egypt (Riegl and Velimirov, 1991; Ibrahim et al., 2011; El-Sorogy et al., 2013; El-Taher and Madkour, 2013), Israel (Loya and Rinkevich, 1980; Riegl and Velimirov, 1991; Loya, 2004; Abelson et al., 2005), Yemen (Alkershi and Menon, 2011), Saudi Arabia (Hanna and Muir, 1990; Badr et al., 2009; Montaser et al., 2010; Ali et al., 2011), Jordan (Al-Najjar et al., 2011), Sudan (Idrisa et al., 2007) and Eritrea (United Nations Development Programme, 2007). Pollution issues in the Red Sea are increasing in severity for several reasons: the Red Sea's small size (458,620 km^2^); the fact that it is bordered by eight countries; and its slow rate of water turnover (Medio et al., 2000).

The sources of pollution in the Red Sea include land-based sources (including urban development, industrial activities, dredging and filling, tourism and agriculture activities), oceanic sources (shipping, fishing, marine traffic and petroleum industries), and atmospheric sources (industries or port activities). Such severe pollution is likely to affect biological life and disturb the Red Sea's natural ecosystems (Regional Organization for the Conservation of the Environment of the Red Sea and Gulf of Aden, 2001). One of the major pollution threats studied is the health of the coral reefs and their ecosystem (Pandolfi et al., 2003; El-Sorogy et al., 2012). Reports on coral reef degradation and the impairment of coral growth and reproduction through algal overgrowth, increased sedimentation and coral disease, have been reported worldwide. Red Sea coral reefs constitute 3.8% of the worlds' coral reefs (PERSGA/GEF, 2004). In an assessment of 21 mangrove sites along the Gulf of Aqaba and the Egyptian Red Sea coastlines, covering ~550 hectares, mangrove degradation was reported from Egypt as a result of oil pollution, tourism, camel grazing and browsing and solid waste accumulation, primarily of plastics (PERSGA/GEF, 2004).

However, equally important is the effect of pollution on microbial life, a topic that has not been well studied in the Red Sea. Two studies have addressed human pathogens along the Red Sea coast (El-Shenawy and Farag, 2005; Ibrahim et al., 2011). In one study, the researchers measured the abundance of saprophytic (SB), salt-tolerant saprophytic (STSB), total coliforms (TC), *Escherichia coli* (EC) and fecal streptococci (FS) in 40 sites along the Egyptian Red Sea coastal waters and in the Aqaba and Suez Gulfs. Of these samples, 91.5% met “European and Egyptian current standards” (El-Shenawy and Farag, 2005). In 2011, TC, EC, and FS in water samples were used as indicators of microbial pollution. This study reported that the Suez Gulf is the most polluted among 194 sites in the Red Sea and Gulf of Aqaba (El-Shenawy and Farag, 2005; Ibrahim et al., 2011).

Conversely, the Red Sea's Egyptian coast has been better studied from perspectives other than the effect of pollution on the microbial life. For example, in 2005, the geochemistry of the sediments and seawater of four Red Sea lagoons were analyzed. The fauna of the Abu Ghoson lagoon was degraded due to the excessive shipping of phosphates, illmenites and feldspars through the port. Safaga Port encounters more than one pollution source causing heavy metal accumulation, including phosphate shipping, adjacent cement industry, land filling, navigation and construction activities, and shipyards (Abd El-Wahab et al., 2005; Mohamed, 2005). Safaga was shown to have the highest concentration of Fe, Pb, Mn, and Zn among the analyzed sites (Abd El-Wahab et al., 2005). The highest concentrations of “P” and “V” were detected in Qusseir Port, and phosphates were detected in high concentrations in its sediments (Mohamed, 2005). Similarly, Hamrawein Port sediments were reported to show distinctive brown coloration, which is characteristic of “P” presence. This is not surprising because Hamrawein Port is one of the oldest harbors for shipping phosphate (Mohamed et al., 2011). Sharm El-Maya is a shallow bay (~6 m deep) that is located in the southern suburb of Sharm El-Sheikh, and its southern reaches are connected to the Red Sea. This bay has been exposed to several oilspill accidents, including the accidental spill of 700 tons of fuel from a cargo ship in 1983 (Roberts and Sheppard, 1988; Khattab et al., 2006), the 1994 oil spill in Sharm El-Sheikh (Pilcher and Abou Zaid, 2000) and the 1999 oil spill accident in Sharm El-Maya (Morsy et al., 2010). All oil spills represent a major threat to the bay ecosystem, including sea grass and coral patches. Additionally, the bay acts as a nursery for commercially valuable fish (Morsy et al., 2010). The combination of the oil spills with the structure and ecosystem of this bay resulted in the entrapment of sediments and oil particles, which caused deleterious effects on coral reef reproduction and the photosynthetic cycle (Loya and Rinkevich, 1980; Al-Halasah and Ammary, 2007).

In addition to the coastal sites of the Red Sea, lakes near the sea can be affected by industrialization. In this study, we analyzed two lakes: Solar Lake at the Gulf of Aqaba (Solar Lake-W) and Saline Lake inside the Ras Muhammed National Park protected area (Saline Lake-RM). Solar Lake-W was selected for this study because it is the only Egyptian Red Sea site where the microbial community has been thoroughly studied. Saline Lake-RM is a petroleum-impacted site that has limited impact from tourism, which allowed us to assess one pollution impact in isolation.

Solar Lake was discovered in 1967 by the workers of the Eilat Nature Reserve (Por, 1968; Eckstein, 1970). They reported the presence of hot water at the bottom of the lake, which was interpreted as hot brines. Later, it was attributed to solar radiation (Eckstein, 1970). When the lake was discovered, it was reported to have the dimensions 80 × 40 m and to be 30 m away from the Red Sea coast, and it was characterized as a meromictic lake (Por, 1968).

Solar Lake is rich in H_2_S and was thought to release high concentrations of H_2_S compared with the production reported from non-polluted waters (Cohen et al., 1975). This has been attributed to the activity of cyanobacterial mats (Cohen et al., 1975). Several strains of cyanobacteria have been isolated from Solar Lake. In 1975, *Oscillatoria limnetica* was isolated from the H_2_S-rich layer of the lake (Cohen et al., 1975). *Cyanobacterial laminites* was detected in different layers of the lake and aided in tracking the age and history of the lake (Cohen et al., 1977). Cyanobacteria control the diurnal cycle of the lake through photosynthesis and O_2_–H_2_S regulation (Jorgensen et al., 1979b). Cyanobacteria anoxygenic photosynthesis is a major contributor to elemental sulfur production and sulfur cycling in the lake (Jorgensen et al., 1979a). *Dactylococcopsis salina*, a gas vacuolated cyanobacterium, was also isolated from the Solar Lake (Walsby et al., 1983). Additionally, a novel species of *Desulfovibrio oxyclinae* was isolated from the Solar Lake cyanobacterial mat. This species was demonstrated to adapt to wide variation in oxygen and sulfide concentrations (Krekeler et al., 1997). Archaeal 16S rDNA analysis demonstrated that halobacteria dominate the archaeal community of the lake and halophilic methanogens were identified in the sulfide- and methane-rich layer (Cytryn et al., 2000).

Few studies have addressed microbial life in Saline Lake-RM. A Gram-negative, haloalkaliphilic and facultative anaerobic bacteria, *Salinivibrio sharmensis*, and *Halomonos sinaiensis* were isolated from Saline Lake (Romano et al., 2007, 2011).

Much work in the Red Sea has focused on visible marine pollution, its various sources and its effects on coral reefs, mangroves and fisheries. However, neglected topics include research on microbial life in Red Sea sediments or water and how pollution affects the distribution and abundance of the microbial communities. Here, we taxonomically identify microbial communities cultured from sediment samples collected at sites that have been impacted by petroleum, industry and tourism.

## Materials and methods

### Site description and sample collection

**Figure 1:**
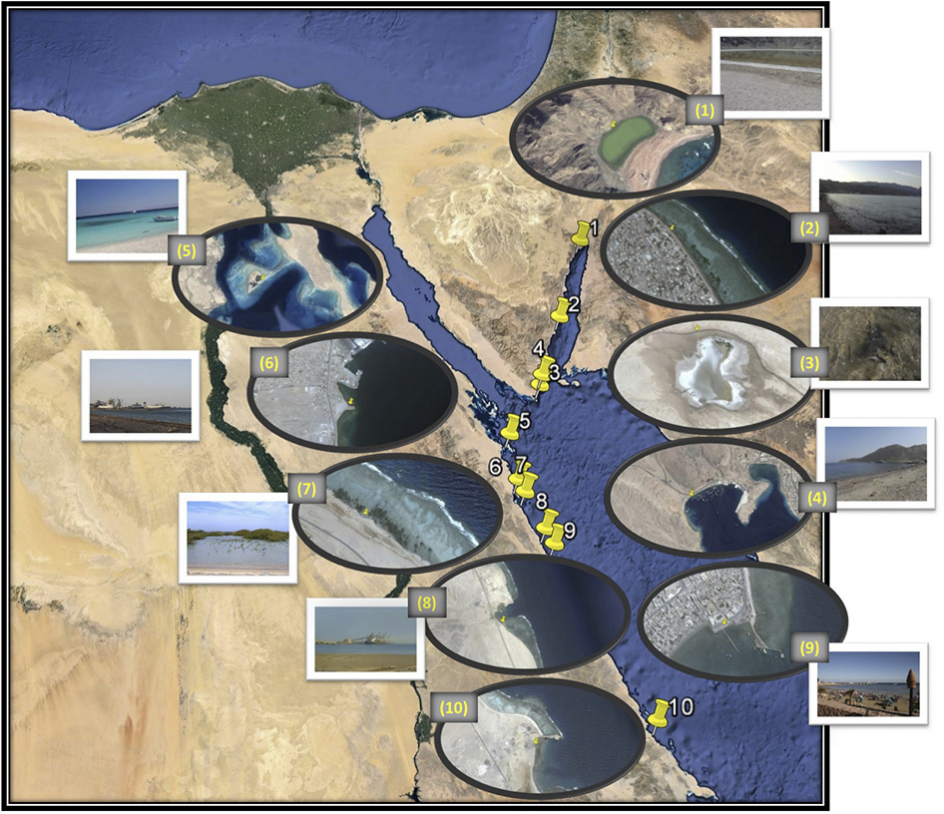
**Map of the locations of the eight coastal sites (numbered 2, 4, and 5–10) and the two lakes (numbered 1 and 3)**. Image generated using Google, Image Landsat Data SIOA, NOAA, U.S. Navy, NGA, GEBCO. The adjacent zoomed image was generated by Image © 2014 DigitalGlobe (sample sites 1, 5, and 6), Image © 2014 CNES/Astrium (sample sites 2 and 4), Data SIOA, NOAA, U.S. Navy, NGA, GEBCO, Image © 2014 CNES/Astrium (sample site 3) and Image © 2014 CNES/Astrium, Data SIOA, NOAA, U.S. Navy, NGA, GEBCO, Image © 2014 TerraMetrics (sample sites 7, 8, 9, and 10). Sample sites: 1. Solar Lake-W, 2. Assala-Dahab, 3. Saline Lake-RM, 4. Sharm El-Maya, 5. Abu-MonkarIsland, 6. Safaga Port (Aluminum), 7. Safaga Port-mangrove, 8. Hamrawein Port, 9. Qusseir port and 10.S-Abu Ghoson port.

Eight coastal sites and two lakes on the Red Sea Egyptian coast were selected for sampling based on the industrial impacts affecting each (Table 1, Figure 1). Six of the ten sites lie on the Red Sea proper, two are on the Sinai Peninsula and two are on the Gulf of Aqaba. The ten analyzed sites included four ports for shipping aluminum (Safaga Aluminum Port), ilmenite (S-Abu Ghoson Port) and phosphate (Qusseir Port and Hamrawein Port), a site previously reported to have suffered oil spills (Sharm El-Maya) and a tourism impacted site (Assala Dahab). Two sites were considered to be non-impacted sites: a protected site (Abu Monkar Island) and a protected mangrove area (Safaga Port-mangrove), which showed oil deposits. The two lakes (Saline Lake-RM and Solar Lake-W) showed different impacts. Saline Lake, which is inside Ras Muhammed, was thought to be a protected area; however we found extensive oil deposits evident in the soft sediments of its dense salt marches. Solar Lake-W is the west shore of the Lake and lies in the direction of the mountains, not toward the rift. The water on the west side of the lake was characterized by green coloration and H_2_S odor, and salt deposits surrounded the lake. Core sampling on the western side of the lake released black fumes (data not shown). Surface sediment samples were collected from the eight sites along the Egypt Red Sea coast in addition to the two lakes in Sinai Peninsula (Table 1, Figure 1). The samples were collected using a basic homemade stainless steel core (5 cm diameter/0.5 m length) and an AMS Multi-Stage Sludge and Sediment Sampler (using one 12″ plastic liner; cat. no. 403.31). The samples were collected near the shore at depths ranging from 0.5 to 1 meter from the sea surface. The middle part of the core (~0.25 m depth) was taken for further analysis to minimize the contamination from the seawater or the shore sand during the on-site handling process.

**Table 1:**
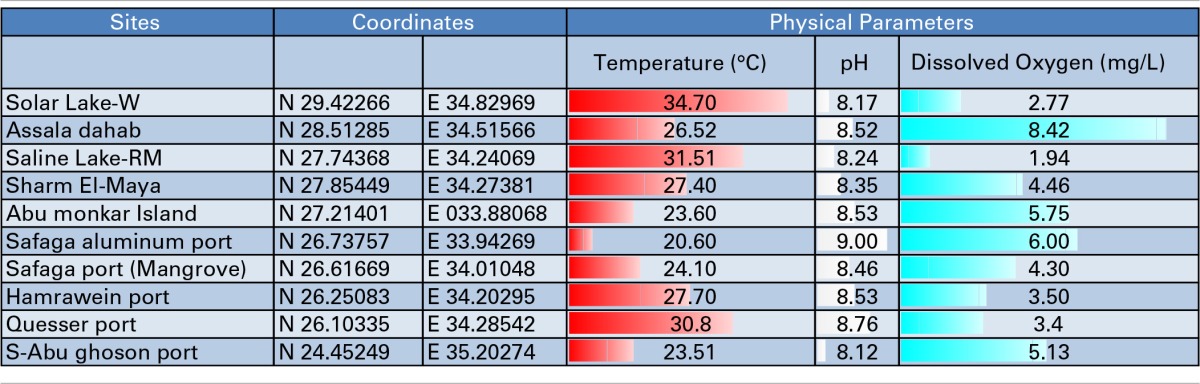
**Samples, Sampling locations and Physical Parameters**.

### Bacterial culturing and DNA preparation

A few grams (~5 g) of the collected sediments was inoculated, on site, directly in 20-mL of freshly prepared Difco™ Marine Broth 2216, using 50 ml falcon tubes to allow for aeration. The cultures were incubated with random mixing for 3 days at room temperature. After delivering the cultures to the lab, the cultures were inverted several times, and 1 ml was taken from each culture for bacterial DNA extraction. The DNA was extracted using QIAamp® DNA Blood Mini Kit (cat no. 51106) following the Protocol for Bacteria in the kit's mini-handbook. The prepared DNA was kept at −80°C for sequencing.

### PCR amplification, 454 pyrotag sequencing and 16S rDNA analysis

For bacterial taxonomic assignment, prepared genomic bacterial DNA was used to amplify the bacterial 16S rDNA hyper-variable regions V6 and V4as previously described (Sogin et al., 2006). The bacterial primers utilized in this study have been described (Siam et al., 2012). The amplicons recovered were subjected to pyrosequencing by 454 GS FLX Titanium technology (454 Life Sciences). V6-V4 reads were deposited in NCBI SRA under the accessions SRR1437688-SRR1437697.

The resources on the Visualization and Analysis of Microbial Population Structures (VAMPS) website, hosted by the Josephine Bay Paul Center, MBL, Woods Hole (http://vamps.mbl.edu/resources/databases.php) were used for the phylogenetic analysis and taxonomic assignment of the reads to major bacterial taxa. Fisher's exact test was used to determine the species/genera that differed significantly in abundance across the different sites (*p* < 0.05, Bonferroni-corrected). The total number of raw reads (no significance test applied) and the number of assigned taxa are presented in Table 2. The significant reads are the reads that passed the cut-off value of the former test. We selected known taxa from these significant reads (significant taxa).

**Table 2:**
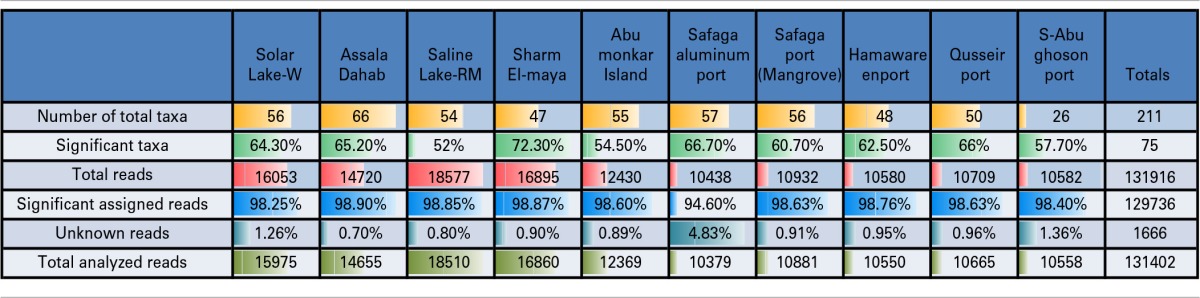
**Pyrotag 16S rDNA data set**.

## Results

### Samples and physical parameters

We attempted to characterize the microbial community along the Egyptian Red Sea coast and two lakes, as illustrated in Figure 1. The coordinates, temperature gradients, pH and dissolved oxygen of the sampling sites are illustrated in Table 1. We started in Solar Lake-W, which is situated near the border of Egypt and Israel on the Gulf of Aqaba (northeastern Egypt), and ended with S-Abu Ghoson Port at the south coast of the Red Sea (southern Egypt). We observed the highest temperatures in the two lakes investigated, Solar Lake-W and Saline Lake-RM, measuring 34.7 and 31.7°C, respectively. The lowest temperature (20.6°C) and highest pH (9.00) were detected in SafagaPort (Aluminum). The remaining sites showed pH ranges of 8.5 ± 0.085. Solar Lake-W and Saline Lake-RM had higher salinity than did the coastal sites (Edwards and Head, 1987; Thompson et al., 2013); the lakes' measured 107.9 and 149.8 ppt, respectively. More variation in the dissolved oxygen was observed in our samples, with Assala-Dahab showing the highest dissolved oxygen saturation (8.42 mg/L), followed by Safaga Port (Aluminum) and Abu-Monkar Island, measuring 6.00 and 5.75 mg/L, respectively (Table 1). Aside from Assala-Dahab, in which the oxygen saturation is considered to be greater than the saturation level of the Red Sea, the dissolved oxygen saturation level in the remaining sites was within the previously reported range (4.8–6.5 ml/L; Institute of Marine Research, 2012).

### Pyrotag 16S rDNA diversity and taxonomic assignments

**Figure 2:**
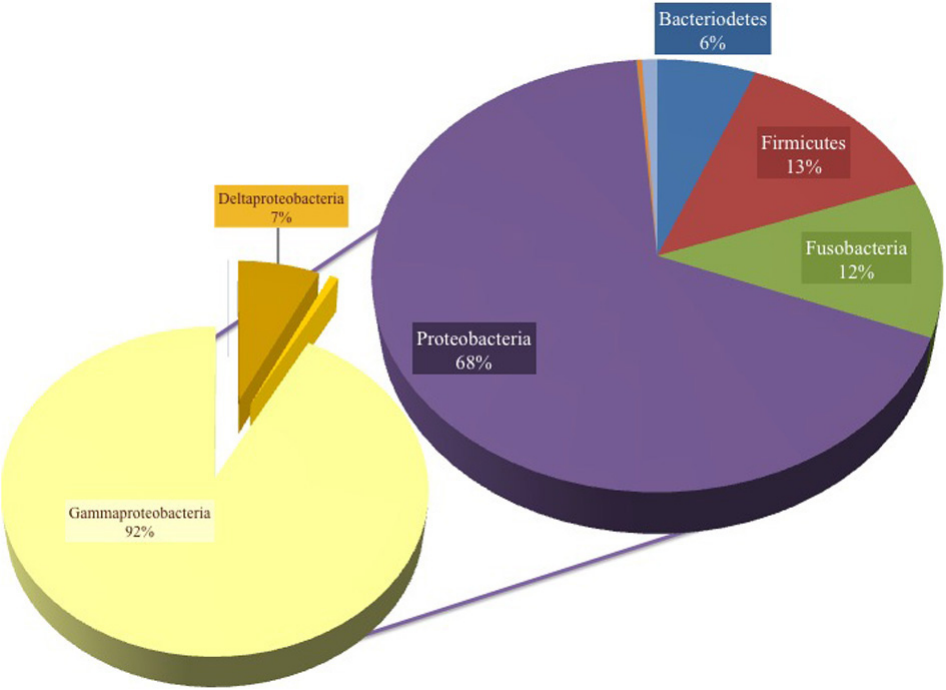
**Pie chart representation of the total cultured phyla in all samples, showing 68% Proteobacteria, 13% Firmicutes, 12% Fusobacteria, 6% Bacteroidetes and unknown bacteria and organism phyla (percent abundance not shown)**. The composition was 92% γ-proteobacteria, 7% δ-proteobacteria and the remaining Proteobacteria were assigned as ε-proteobacteria.

A total of 131,916 reads were generated from the cultured Red Sea coastal sediments and the two lakes using pyrotag sequencing. The taxonomic assignment of the reads to major bacterial genera detected 211 different genera. Following significance testing, we concluded that only 75 genera were significantly detected; these were represented by a total of 131,402 reads. Only 1.3% of these were unassigned reads, including both unassigned bacteria and unassigned organisms. The label “unassigned organism” indicates a taxon of unknown bacteria, archaea or eukarya (Table 2). We compared the diversity of bacterial phyla reads across the sites. Using the media and conditions described in the materials and methods section, five major bacterial phyla were cultured from the 10 sites sampled, predominantly Proteobacteria (68%), followed by Firmicutes (13%), Fusobacteria (12%), Bacteriodetes (6%) and Spirochetes (0.03%) (Figure 2). Members of Proteobacteria included γ-proteobacteria (92%), followed by δ–proteobacteria (7%) and ε-proteobacteria (1%) (Figure 2, Table 3). Very low proportions of unassigned organisms were detected across sites (0.9% collectively).

**Table 3:**
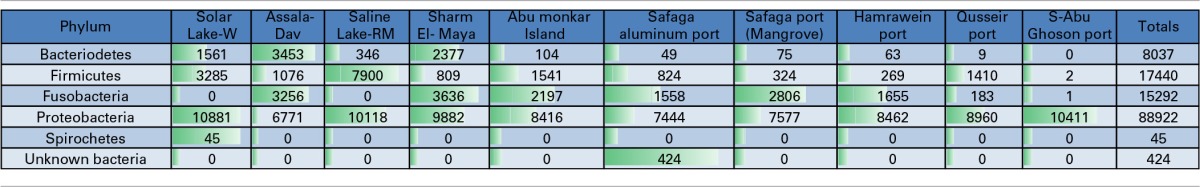
**Total number of reads assigned at the phylum level in Red Sea coastal samples and two lakes**.

Proteobacteria-assigned reads predominated at all of the sampled sites. No other phyla were significantly detected in S-Abu Ghoson Port. Fusobacteria were not detected in the two lakes. Firmicutes represent the second most abundant phylum in the two lakes and Qusseir Port. Bacteriodetes, Firmicutes and Fusobacteria were detected in the remaining coastal samples, but in varying abundances. For example, Assala-Dahab showed the highest incidence of Bacteriodetes (43/23.6% total Bacteriodetes/total culture), followed by Sharm El-Maya (30/14.1% total Bacteriodetes/total culture). Sharm-El-Maya showed the highest incidence of Fusobacteria (23.7%), followed by Assala-Dahab (21.3%). Fusobacteria was the second most abundant phylum in all of the sites, except Qusseir Port, S-Abu Ghoson Port and the two lakes. Unassigned organisms were the second most abundant phylum in S-Abu Ghoson Port. Interestingly, all reads for Spirochetes were detected in Solar Lake-W. A significant number of reads (424) representing 0.3% of the total analyzed reads were assigned as unknown bacterial phylum; these were only detected in Safaga Port (Aluminum) (Table 3).

### Bacterial consortia along the red sea coast

An average of 34 ± 1.6 reads were assigned to the remaining seven Red Sea Coast samples, with rare reads constituting 28 ± 1.5. The S-Abu Ghoson Port reads were unique from the other coastal sites: 15 bacterial reads were identified, of which 11 were considered rare. Unknown species of Photobacterium (50%) and *Photobacterium halotolerans* (24%) predominated among the bacterial-assigned reads in S-Abu Ghoson Port. Unassigned *Vibrio* spp. predominated among the reads in the remaining Red Sea Coast samples (53.4%), followed by *Propionigenium maris* (15.4%). Qusseir Port and S-Abu Ghoson Port were exceptions (Table S1).

Table 4A shows a preliminary taxonomic assignment of V6-V4 reads to the previously reported pathogenic bacteria (strictly infecting humans). Note that the V6-V4 reads are relatively short for assigning taxa at the species level. However, we observed that *Clostridium botulinum* and three assigned species of *Vibrio* represented the pathogenic bacteria detected in the cultures of these Red Sea sites. In total, *Vibrio parahaemolyticus* was the most abundant pathogenic bacterium (48.5/0.67% of the total pathogenic bacteria/total reads), followed by *Clostridium botulinum* (35.8/0.5% of the total pathogenic bacteria/total reads). The pattern of the pathogenic bacteria (distribution and abundance) in the Solar Lake-W was different from the remaining sites, as *Clostridium botulinum* was detected only at this site. As expected, the taxonomic assignment of Saline Lake-RM reads did not match any known pathogenic bacteria, followed by Abu-Monkar Island and Assala-Dahab, which showed the lowest abundance of reads to pathogenic bacteria (0.8/0.1 and 2.2/0.3% of the total detected pathogenic bacteria/the site's total cultured bacteria, respectively).

**Table 4:**
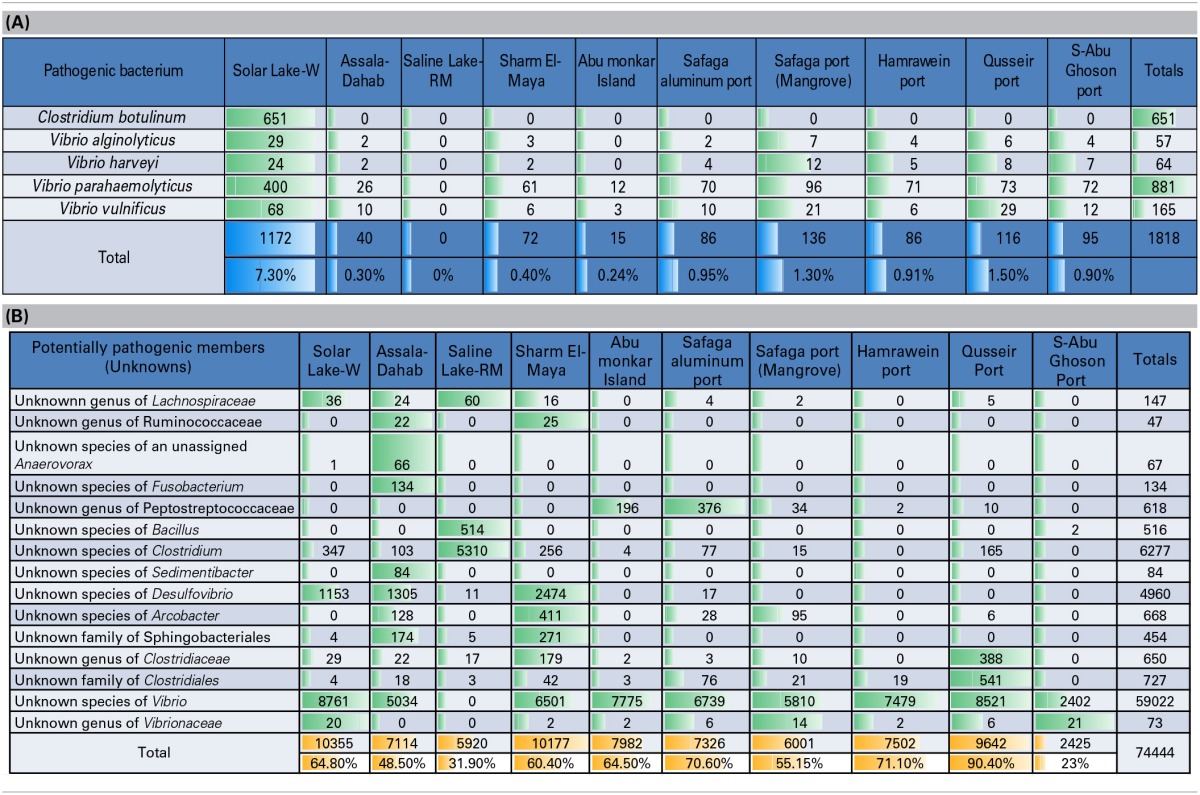
**(A) Preliminary assignment of total number of reads to known human pathogens, (B) Total number of reads assigned to potential pathogens (previously reported genera/species included human pathogens and non-pathogens)**.

We also detected reads for which previously identified genera/species are reported to include both pathogenic and non-pathogenic members. We refer to these as potential pathogens (Table 4B). Our culture approach has detected these potentially pathogenic bacteria, including five unknown families of Lachnospiraceae, Ruminococcaceae, Peptostreptococcaceae, Clostridiaceae and Vibrionaceae. Additionally, eight unknown species of unassigned *Anaerovorax, Fusobacterium, Bacillus, Clostridium, Sedimentibacter, Desulfovibrio, Arcobacter*, and *Vibrio*. Additionally, two unknown orders of Sphingobacteriales and Clostridiales were detected, and we categorized them as potentially pathogenic bacteria.

### Solar lake-W and saline lake-RM bacterial consortia

**Figure 3:**
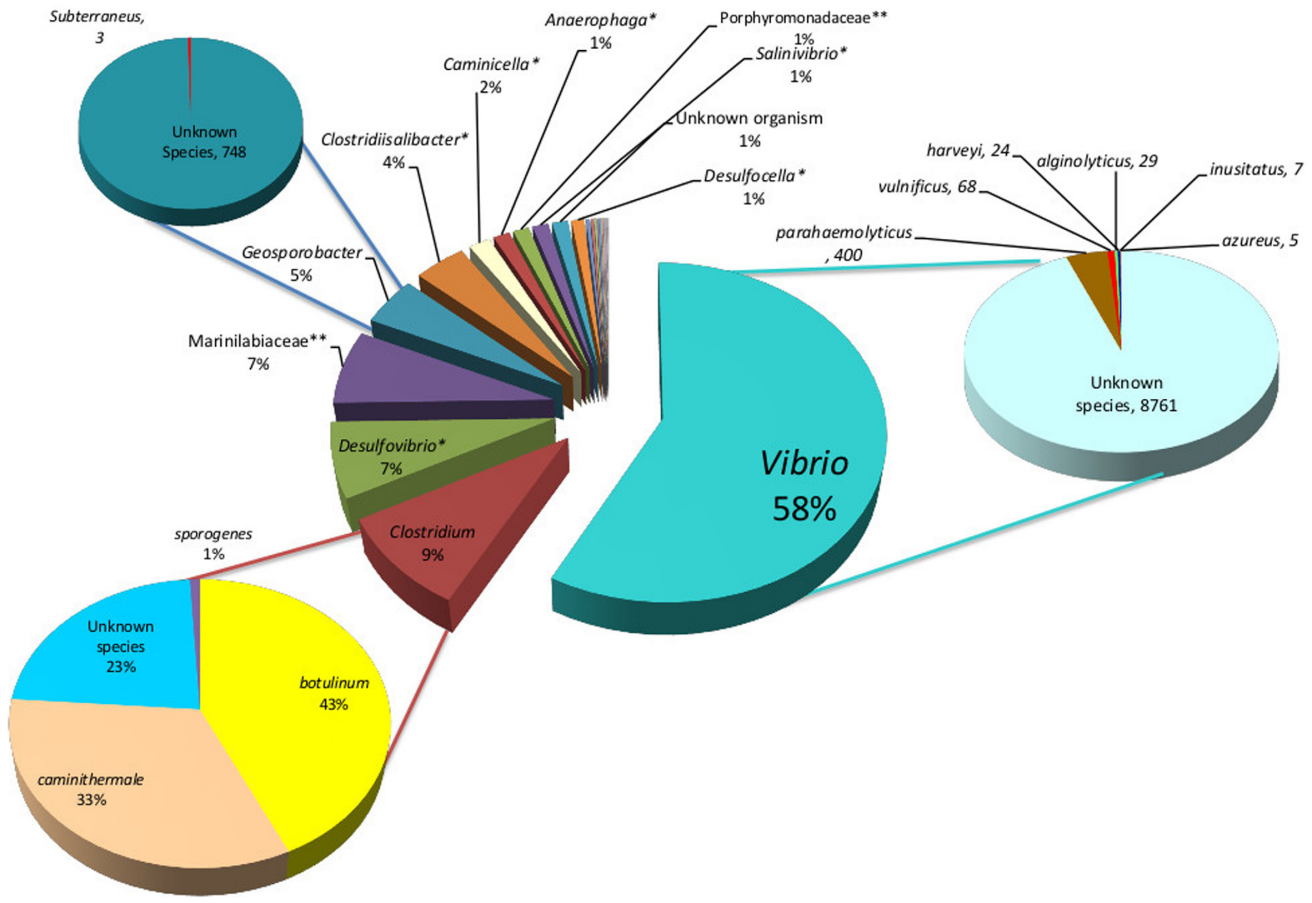
**Solar Lake West genera are presented in the middle pie chart, predominated by *Vibrio* (58%), *Clostridium* (9%), unknown species of *Desulfovibrio* (7%) and Geosporobacter (5%)**. The *Vibrio, Desulfovibrio* and Geosporobacter are predominantly unknown species. The predominant *Clostridium* species is *C. botulinum* (43%). ^*^unknown species;^**^unknown genus.

In total, 36 and 28 bacterial reads were cultured and amplified from the Solar Lake-W and Saline Lake-RM, respectively. Four bacterial reads were unique to these two lakes, including reads assigned to *Orenia marismortui* and unknown species of Caloranaerobacter, Clostridiisalibacter and Halomonas. Eight and nine reads were unique to Solar Lake-W and Saline Lake-RM, respectively (Table 5). Of the 36 bacterial reads cultured from Solar Lake-W, 21 are considered rare bacterial reads (less than 1%). Unknown species of *Vibrio* dominated the cultured community (55%). The remaining reads constituted six species of *Vibrio* (*Vibrio parahaemolyticus*-2.5%; Figure 3). Surprisingly, 9% of the culture was assigned to the genus *Clostridium*, with 4.1% assigned as *Clostridium botulinum*. Seven percent of the culture was assigned to *Desulfovibrio* and 4% to *Clostridibacter*. Conversely, an unknown genus of Marinlabiacae and *Geosporobacter* genus represented 7% and 5% of the total bacterial reads of this lake, respectively (Figure 3).

**Table 5:**
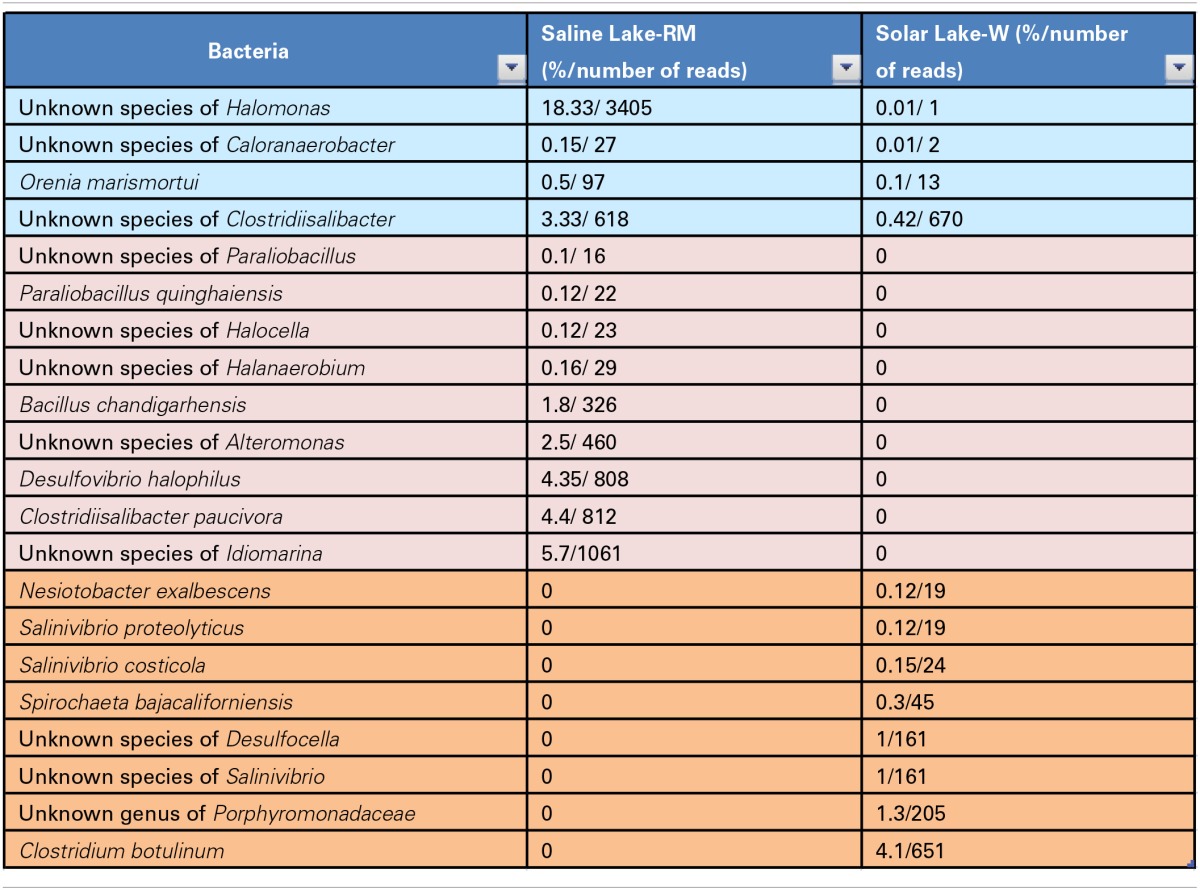
**Bacteria identified only in the two lakes**.

Of the 28 bacterial reads in the Saline Lake-RM, 17 were considered to be rare bacterial taxa (less than 1%). Saline Lake-RM was dominated by an unknown species of *Clostridium* (29%), followed by an unknown species of *Marinobacter* (24%), an unknown species of *Halomonas* (18%), and an unknown species of *Idiomarina* (5.7%). Only six bacterial reads were assigned at the species level: *Clostridiisalibacter paucivorans* (*4*.3%), *Desulfovibrio halophilus* (4.3%), *Bacillus chandigarhensis* (1.8%), *Orenia marismortui* (0.5%), *Paraliobacillus quinghaiensis* (0.12%), and *Clostridium caminithermale* (*0.01%*).

**Figure 4:**
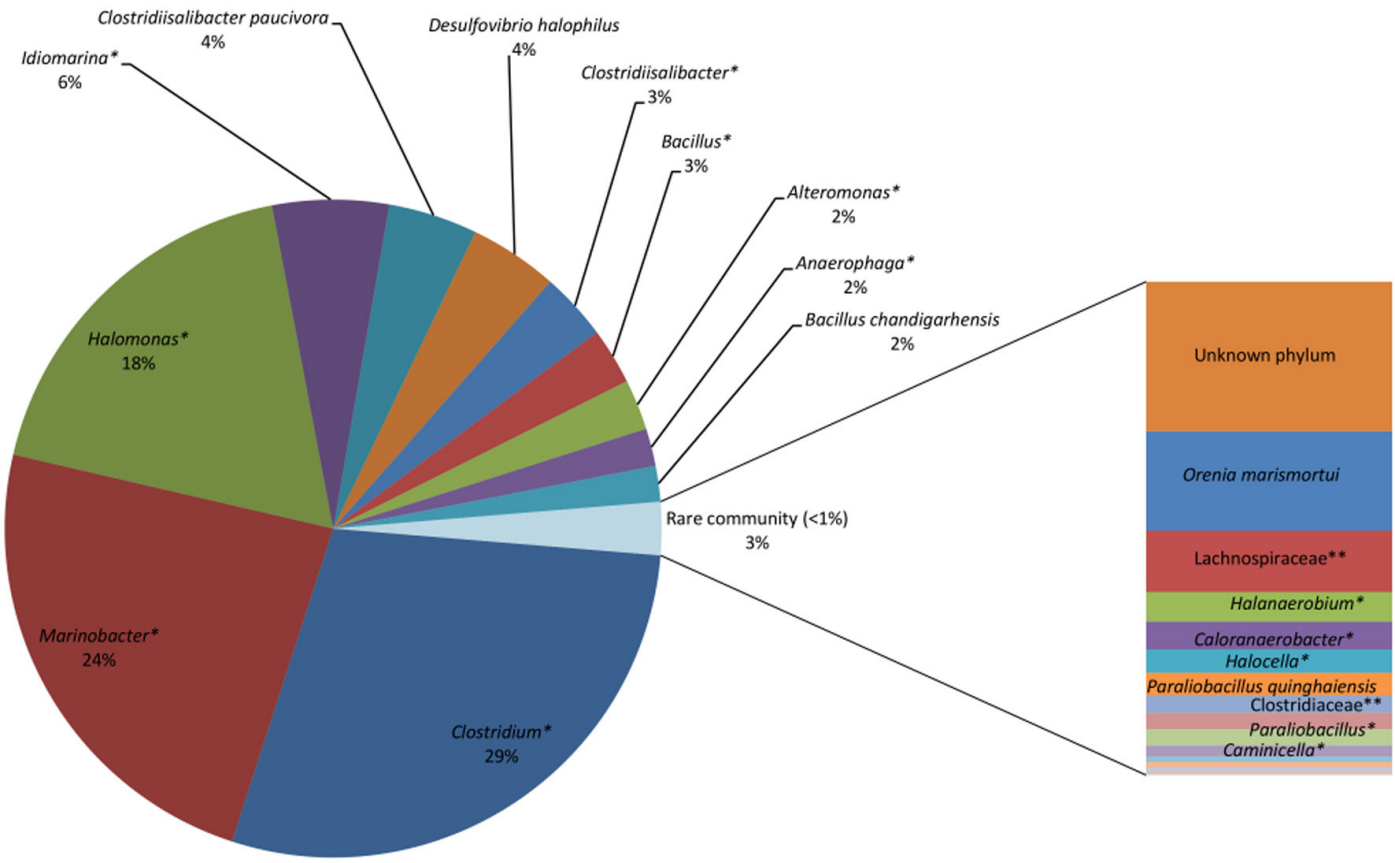
Saline Lake bacterial consortia showing predominantly unknown species, including unknown species (^*^) of *Clostridium* (29%), Marinobacter (24%), *Halomonas* (18%), *Idiomarina* (6%), Clostridiisalibacter (3%), Bacillus (3%), and *Alteromonas* (2%). Known species identified include *Clostridiisalibacterpaucivora* (4%), *Desulfovibrio halophiles* (4%) and *Bacillus chandigarhensis* (2%). ^*^Unknown species

Four more unknown species were significantly detected: unknown species of Clostridiisalibacter, Bacillus, Alteromonas and Anaerophaga account for 3.3, 2.8, 2.5, and 1.8% of assigned reads, respectively. The rare bacterial taxa (below 1%) include two species, nine genera, two families and two orders (Figure 4).

## Discussion

We analyzed the microbial community in sites that have been impacted by, land-based, oceanic and atmospheric pollution sources along the Red Sea coast. Additionally, we assessed the microbial community in two lakes on the Sinai Peninsula: Solar Lake-W and the Saline Lake-RM, which are believed to have seeps from the Red Sea (Aharon et al., 1977). Following sediment cultures, we used the V6-V4 hypervariable region and amplified a significant number of pyrotags; 131,916 16S rDNA reads were obtained, of which 211 were assigned to major bacterial taxa. We analyzed the 75 significant taxa detected (131,402 reads) and grouped them into two major categories: human pathogens and oil-degrading bacteria.

We used a culture media that allows the cultivation of heterotrophic marine bacteria. Therefore, this study examines a portion of the bacterial community in these environments. Our cultured marine surface sediment samples had several taxa in common. For example, γ-proteobacteria dominated all of the cultured bacteria in the10 sampled sites. Several previous culture-independent approaches showed a significant dominance of γ-proteobacteria in marine sites (Liao et al., 2009), followed by Firmicutes, Fusobacteria and Bacteriodetes. A previous study identified Firmicutes and Bacteriodetes from RasMuhammed sponges using a culturing approach (Radwan et al., 2010; Aboul-Ela et al., 2012). Fusobacteria have previously been detected in the Red Sea; however, they were detected in deep sediments of a brine pool (Siam et al., 2012). Most species of Fusobacteria and Bacteriodetes are anaerobes. Fusobacteria have been cultured from surface sediments samples of the Wadden Sea, Germany, under strict anaerobic conditions (Köpke et al., 2005). Note that strict anaerobic conditions were not implemented in our culture conditions; however, anaerobic bacteria such as Fusobacteria, Bacteriodetes and Clostridium were detected. Because our culture conditions are most likely enriched for aerobes, it is likely that such anaerobes are more abundant *in situ*. This finding suggests that we may have cultured the predominant phyla in our studied sites; however, rare taxa are as important in the microbial community. This imposes a limitation on our study, in that it strictly focuses on the numerically dominant bacterial taxa. A culture-independent approach would allow the study of the entire bacterial community.

Several human pathogens were detected in our Red Sea samples, including known *Vibrio* and *Clostridium* species. *Vibrio* species are naturally detected in marine environments (Johnson et al., 2010). *Vibrio* spp. have been found to dominate “plastisphere” (i.e., plastic marine debris; Zettler et al., 2013). In this culture-independent study, 10 different *Vibrio* species were detected and were dominated by an unknown species of *Vibrio*. Surprisingly, our culture-dependent approach detected 28 *Vibrio* species; of these, 12 were significantly detected in our cultures, which were dominated by an unknown species of *Vibrio*. This “marine *Vibrio* phenomenon” was detected at all of our sites except Saline Lake-RM. In addition to the striking dominance of this unknown species of Vibrio, three of the remaining 11 known *Vibrio* species were human pathogens. Several *Vibrio* species have been reported to cause gastrointestinal, skin and other infections (Thompson et al., 2004), including *V. parahemolyticus, V*. *vulnificus* and *V. alginolyticus*. Note that *V. vulnificus, V. splendinus and V. sinaloensis* were not detected in the “plastisphere” *Vibrio* community (Zettler et al., 2013). *V. shilonii*, which was detected in our cultures, is known to cause coral bleaching (Banin et al., 2000; Thompson et al., 2004). *V. fortis* and *V. harveyi* may contribute to coral bleaching (Thompson et al., 2004). Solar Lake-W was most dominated by pathogenic bacteria, which constituted 7.3% of the Solar Lake-W bacterial culture; these were primarily *Vibrio parahaemolyticus*. One limitation in our study is the short read length of the V6-V4 region, which does not provide optimal resolution at the species level. We therefore cannot assign taxa to the species level based solely on V6-V4 16S rRNA. However our results imply that the “marine *Vibrio* phenomenon” may pose a pathogenicity risk for human and/or marine life.

Similarly, other human pathogens were detected in our sediment samples. Most importantly, *Clostridium botulinum* was uniquely and significantly detected in Solar Lake-W. Clostridial members are known for their ability to survive under harsh conditions through spore formation. *Clostridium botulinum* outbreak detection includes the detection of spores from the contaminated environment, which may include soil and aquatic environments (e.g., marine sediments; Neimanis and Speck, 2012). This may explain the detection of strict anaerobes under our aerobic culture conditions: we could have isolated bacterial DNA from bacterial spores. Additionally, it is not uncommon to detect *Clostridium* in marine sediments, as this is one of its natural habitats(Neimanis and Speck, 2012). However, *C. botulinum* is a serious human pathogen (Neimanis and Speck, 2012). Botulism was first reported in 1991 from a traditional Egyptian salted raw fish, known as “faseikh” (Weber et al., 1993). Since then, no botulism cases have been reported in Egypt (Horowitz, 2010). In a 2011 study *C. botulinum* was isolated from food samples in Assiut, Egypt, but no botulism cases were reported (Ahmed et al., 2011).

In contrast to all of the other sites, Saline Lake-RM had no cultured/detected known pathogenic *Vibrio* or *Clostridium*. The bacterial community in Saline Lake-RM is predominated by a different and unknown bacterial community that is more likely to play a role in hydrocarbon metabolism. Other unknown bacterial families, genera and species were detected in different samples along the Red Sea coast (Table 4B). Related members of these bacterial groups were shown to be human pathogens. Due to the lack of assignments of bacterial reads to known families, genera or species, we may consider them potential pathogens. Further analysis on these groups should be performed to identify their pathogenic potential.

It was not surprising to detect oil-degrading bacteria along the Red Sea, particularly at industrialized sites. However, the oil-degrading bacterial consortia detected in the Red Sea coastal samples were distinct from those detected in the two lakes. *Propionigenium maris, Psychrilyobacter*, Tepidibacter and Photobacterium were mainly detected in the Red Sea coastal samples. *Propionigenium maris* is a marine debrominating bacteria (Watson et al., 2000). This species was one of the most abundant species in most of the analyzed sites. *Psychrilyobacter* produces H2 and acetate and has the ability to degrade hexahydro-1,3,5-trinitro-1,3,5-triazine and octahydro-1,3,5,7-tetranitro-1,3,5,7-tetrazocine, two nitramine explosives (Zhao et al., 2009). Tepidibacter was isolated from an oil field in China (Tan et al., 2012). Photobacteriumspecies show mercuric resistance (Reyes et al., 1999), are stable in biodiesel production at high methanol concentrations (Yang et al., 2009) and have high oil emulsification activity (Ryu et al., 2006). A bacterial consortium constituting 21 taxa was unique to the two lakes (Table 5). Additionally, three unknown species of Marinobacter and Bacillus were detected in Saline Lake-W only, and Anaerophaga was detected in both lakes. The unknown species of Marinobacter was the second most abundant taxa in Saline Lake-RM (24%). Previous studies have identified several strains of Marinobacter as oil-degrading bacteria that can degrade aliphatic hydrocarbons under oxic conditions (Cohen, 2002; Duran, 2010). An unknown species of *Idiomarina* was also uniquely identified in Saline Lake-W (5.7%). A strain of crude oil-degrading bacteria *Idiomarina xiamenensis* was isolated from surface water enriched in crude oil (Wang et al., 2010, 2011). Interestingly, the sulfate-reducing bacterium *Desulfovibrio halophilus* was isolated for the first time from the Solar Lake (Caumette et al., 1991). However, in our study, we have only detected it in Saline Lake-RM. This species was also detected in brine stratal water of an oil field (Welsh et al., 1996). This bacterium is known to accumulate organic solutes under high salt conditions (Welsh et al., 1996), such as those in Saline Lake-RM. We identified an oil-degrading bacterial consortium in Saline Lake-RM. *Anaerophaga* (detected in the two lakes only) was present in blackish-oily sedimentary residues in an oil separation tank (Denger et al., 2002; Schink, 2010). It is worth noting that all of the previous studies on Solar Lake isolated specific cyanobacteria and analyzed the cyanobacterial mats (Krumbein et al., 1977; Jorgensen et al., 1979b; Teske et al., 1998; Wieland and Kühl, 2000). Strikingly, our approach did not detect or assign any of the reads to cyanobateria in the Solar Lake culture. Note that cyanobacteria were not detected in uncultured sediments (data not shown). Taken together with our results, the studies conducted on Solar Lake West near Taba/Eilat between its discovery in 1967 and the last reported study in 1983 suggest that the microbial community in this lake has varied greatly during the past 20 years. This finding draws attention to the importance of microbial studies in monitoring and conserving marine environments.

This study molecularly characterized cultured microbial consortia along Egypt's Red Sea coast, with a focus on industrialized sites. Our results demonstrate the dominance of *Vibrio* spp. (human pathogens, coral pathogens and predominantly unknown species), common marine bacteria, hydrocarbon-degrading bacteria and other human pathogenic bacteria. The oil-degrading bacterial consortia were distinctly unique in the Red Sea coast compared with the two lakes sampled, suggesting different hydrocarbon exposures in these two ecosystem types. Additionally, the human pathogen consortia were dominated by *Vibrio* spp., which is different from the Saline Lake-W, where no known bacterial pathogens were detected. This study provides preliminary evidence for the use of bacterial consortia to assess the impact of industrialization on marine environments.

### Conflict of interest statement

The authors declare that the research was conducted in the absence of any commercial or financial relationships that could be construed as a potential conflict of interest.

## Supplementary material

Table S1 **Bacterial communities comprisingmore than 0.1% in each site are represented**.
