# Supplementary material for: Egypt's Red Sea coast: phylogenetic analysis of cultured microbial consortia in industrialized sites
Source: Front Microbiol. 2014 Aug 11;5:363. doi: 10.3389/fmicb.2014.00363 (PMC4127681; doi:10.3389/fmicb.2014.00363)
Supplement: Table S1 — Bacterial communities comprisingmore than 0.1% in each site are represented. [file DataSheet1.PDF]

Name of the unrare taxa	Name of the site	Solar lake-W	Assala-Dahab	Saline lake-RM	Sharm El-Maya	Abu Monkar Island 3	Safaga Aluminum Port	Safaga Port (Mangrove)	Hamrawein Port	Qusseir Port	S-Abu Ghoson Port
	Number of taxa	36	41	28	34	30	38	34	29	33	15
	Rare taxa	21	34	17	25	23	31	29	25	27	11
Unknown Species of *Vibrio*		0.545755	0.341984	NA	0.384788	0.625503	0.645622	0.531419	0.7069	0.795686	0.226989
Propionigenium maris		NA	0.209783	NA	0.213673	0.129928	0.140832	0.218604	0.153119	0.0127	NA
Unknown Species of *Fusibacter*		NA	0.024592	NA	NA	0.102333	0.025675	NA	NA	NA	NA
Unknown Species of *Psychrilyobacter*		NA	NA	NA	NA	0.045776	NA	0.035214	NA	NA	NA
Unknown Species of *Photobacterium*		NA	NA	NA	0.011423	0.020837	0.025867	0.109119	0.059641	NA	0.503686
Unknown genus of Peptostreptococcaceae		NA	NA	NA	NA	0.015768	0.036022	NA	NA	NA	NA
Unknown genus of Marinilabiaceae		0.070952	0.215829	NA	0.124534	NA	NA	NA	NA	NA	NA
Unknown Species of *Desulfovibrio*		0.071825	0.088655	NA	0.213673	NA	NA	NA	NA	NA	NA
Unknown Species of *Geosporobacter*		0.046596	0.016304	NA	NA	NA	NA	NA	NA	NA	NA
Unknown family of Sphingobacteriales		NA	0.011821	NA	0.01604	NA	NA	NA	NA	NA	NA
Unknown Species of *Tepidibacter*		NA	NA	NA	NA	NA	NA	0.021495	0.022401	0.026613	NA
Unknown genus of Clostridiaceae		NA	NA	NA	0.010595	NA	NA	NA	NA	0.036231	NA
Unknown Species of *Clostridium*		0.021616	NA	0.285837	0.015152	NA	NA	NA	NA	0.015408	NA
Unknown Species of *Clostridiisalibacter*		0.041737	NA	0.033267	NA	NA	NA	NA	NA	NA	NA
Unknown Species of *Anaerophaga*		0.013269	NA	0.018356	NA	NA	NA	NA	NA	NA	NA
Unknown organism		0.012646	NA	NA	NA	NA	NA	NA	NA	NA	0.013608
Photobacterium leiognathi						0.018262					
Unknown family of Clostridiales										0.050518	
Unknown bacterial Phylum							0.040621				
Unknown Species of *Pseudoalteromonas*							0.01552				
Unknown Species of *Marinobacter*				0.235399							
Unknown Species of *Halomonas*				0.183291							
Unknown Species of *Idiomarina*				0.057114							
Clostridiisalibacter paucivora				0.04371							
Desulfovibrio halophilus				0.043495							
Unknown Species of *Bacillus*				0.027669							
Unknown Species of *Alteromonas*				0.024762							
Unknown Species of *Anaerophaga*				0.018356							
Bacillus chandigarhensis				0.017549							
Photobacterium halotolerans											0.23918
Unknown Species of *Arcobacter*					0.024327						
Clostridium botulinum		0.040553									
Clostridium caminithermale		0.031271									
Vibrio parahaemolyticus		0.024917									
Unknown Species of *Caminicella*		0.017255									
Unknown genus of Porphyromonadaceae		0.01277									
Unknown Species of *Desulfocella*		0.010029									
Unknown Species of *Salinivibrio*		0.010029									
